# Supplementary material for: Tead1a Initiates Transcriptional Priming Through the TEAD1a/YAP‐Notch1‐Spi1/Cebpα Axis to Promote Neutrophil Fate
Source: Adv Sci (Weinh). 2025 Aug 28;12(41):e05441. doi: 10.1002/advs.202505441 (PMC12591127; doi:10.1002/advs.202505441)
Supplement: Supplementary file 4 — Supplementary Dataset [file ADVS-12-e05441-s004.zip › 3-3.zip!/3-3/02-3.cluster/cluster7/Enrich/ego_all.pdf]

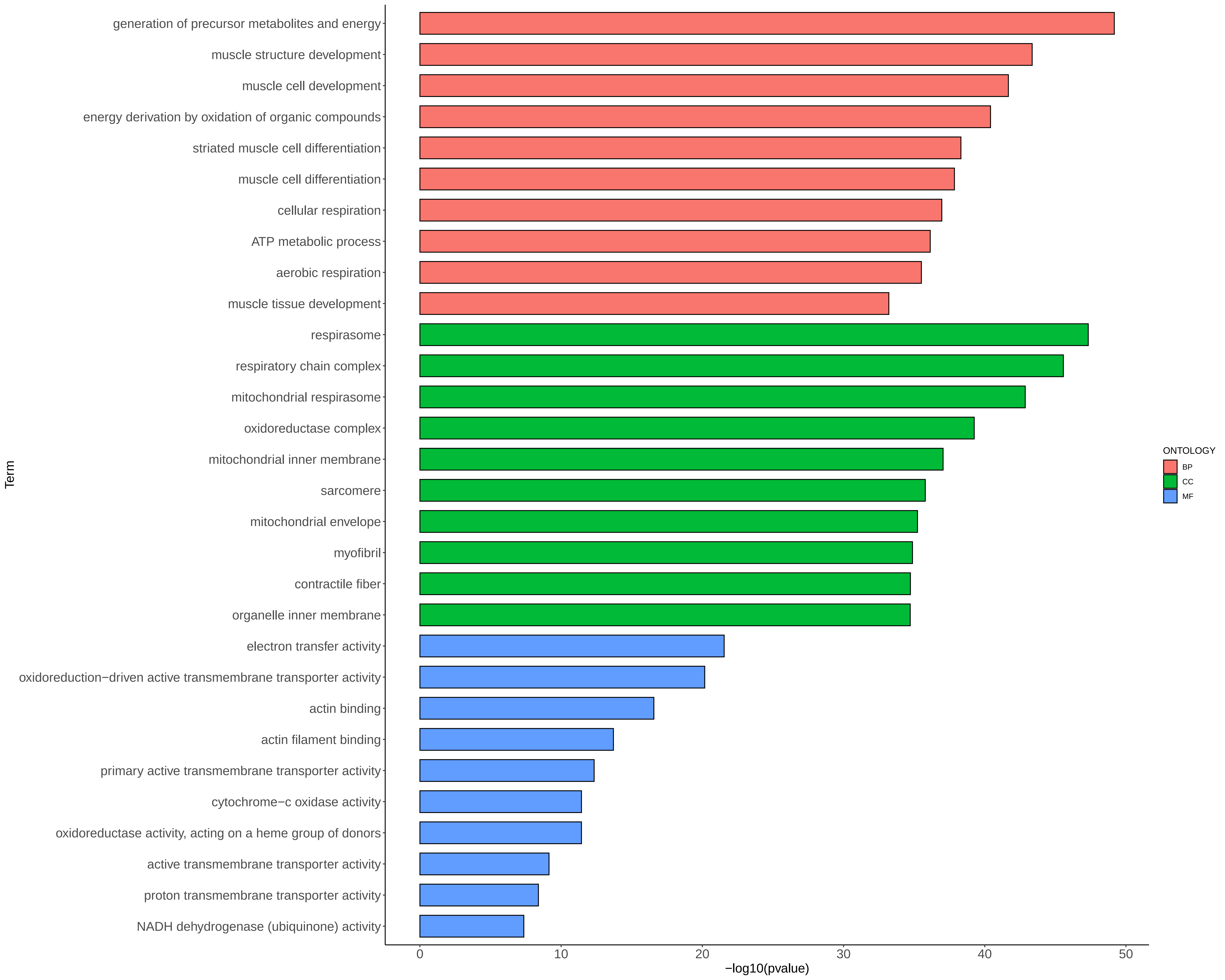
generation of precursor metabolites and energy
muscle structure development
muscle cell development
energy derivation by oxidation of organic compounds
striated muscle cell differentiation
muscle cell differentiation
cellular respiration
ATP metabolic process
aerobic respiration
muscle tissue development
respirasome
respiratory chain complex
mitochondrial respirasome
oxidoreductase complex
mitochondrial inner membrane
sarcomere
mitochondrial envelope
myofibril
contractile fiber
organelle inner membrane
electron transfer activity
oxidoreduction−driven active transmembrane transporter activity
actin binding
actin filament binding
primary active transmembrane transporter activity
cytochrome−c oxidase activity
oxidoreductase activity, acting on a heme group of donors
active transmembrane transporter activity
proton transmembrane transporter activity
NADH dehydrogenase (ubiquinone) activity
Term
0
10
20
30
40
50
−log10(pvalue)
ONTOLOGY
BP
CC
MF
